# Notch Inhibits Yorkie Activity in *Drosophila* Wing Discs

**DOI:** 10.1371/journal.pone.0106211

**Published:** 2014-08-26

**Authors:** Alexandre Djiane, Sophie Zaessinger, A. Burcu Babaoğlan, Sarah J. Bray

**Affiliations:** 1 Department of Physiology Development and Neuroscience, University of Cambridge, Cambridge, United Kingdom; 2 IRCM, Institut de Recherche en Cancérologie de Montpellier, Montpellier, France; INSERM, U896, Montpellier, France; Université Montpellier1, Montpellier, France; Institut régional du Cancer Montpellier, Montpellier, France; MRC, University College of London, United Kingdom

## Abstract

During development, tissues and organs must coordinate growth and patterning so they reach the right size and shape. During larval stages, a dramatic increase in size and cell number of *Drosophila* wing imaginal discs is controlled by the action of several signaling pathways. Complex cross-talk between these pathways also pattern these discs to specify different regions with different fates and growth potentials. We show that the Notch signaling pathway is both required and sufficient to inhibit the activity of Yorkie (Yki), the Salvador/Warts/Hippo (SWH) pathway terminal transcription activator, but only in the central regions of the wing disc, where the TEAD factor and Yki partner Scalloped (Sd) is expressed. We show that this cross-talk between the Notch and SWH pathways is mediated, at least in part, by the Notch target and Sd partner Vestigial (Vg). We propose that, by altering the ratios between Yki, Sd and Vg, Notch pathway activation restricts the effects of Yki mediated transcription, therefore contributing to define a zone of low proliferation in the central wing discs.

## Introduction

Correct tissue development involves the successful co-ordination of growth and patterning mechanisms. One tissue that lends itself to the study of such co-ordination is the wing imaginal disc in *Drosophila*. The wing develops from groups of epithelial cells, which are specified during embryogenesis. When the embryo hatches, these wing imaginal discs consist of about 10 cells and then, through extensive proliferation during larval life, they reach several thousands cells by the time of metamorphosis. At the same time as they undergo this spectacular increase in cell number, they become fully patterned. Their proliferation, growth and patterning are regulated by the activity of several conserved signaling pathways, including Notch [1], and it is important to understand how these pathways co-operate to generate a wing of the right shape and size.

In wing imaginal discs, Notch controls tissue growth and cell proliferation through the regulation of a complex network of genes whose products act both cell autonomously (e.g. Myc, DIAP1, CycE) and cell non-autonomously (e.g. secreted ligands of the Wnt and Upd family) [2]–[5]. Strikingly, while Notch is required throughout the wing disc for tissue growth, the effects of its over-activation on cell proliferation are most evident at the periphery of the wing disc, outside of the central region or pouch [6], [7]. Activation of Notch involves two proteolytic cleavages, which release the intracellular part of the receptor (Nicd). Nicd then enters the nucleus, binds to the transcription factor Suppressor of Hairless (Su(H)) to turn on the transcription of target genes [8]. A major output of Notch activation is therefore the transcriptional up-regulation of responsive genes. The differential effects on growth in the wing appear to be the consequence of an additional set of genes, including *scalloped* and *vestigial*, which are upregulated by Notch in the central wing-pouch [9]–[12] where they prevent the expression of proliferation promoting genes [5].

At the same time as requiring Notch, the epithelial cells also depend on the Salvador/Warts/Hippo (SWH) pathway to regulate their proliferative potential. Defects in this pathway cause dramatic tissue overgrowth. At its core the SWH pathway contains a kinase-cassette, consisting of Warts and Hippo, whose activation results in the phosphorylation of the transcription co-activator Yorkie (Yki; YAP/TAZ in vertebrates). As a consequence Yki/YAP is excluded from the nucleus and is thus prevented from acting with its DNA-binding transcription factor partners. The latter include Scalloped/TEAD, Homothorax, p53 and, by binding with these, Yki/YAP promotes the expression of pro-survival and pro-proliferation genes [13]–[16]. One of the best characterized Yki targets is the gene *expanded (ex)* [15]. As *ex* encodes a cortical protein of the FERM family, which is itself implicated in activation of SWH pathway [17], the gene is part of a negative feedback loop regulating Yki activity.

One critical role of Notch in the wing disc is that it governs the expression of Sd and the Sd-binding partner Vestigial (Vg) to specify regions of the wing disc with distinct proliferative potential [3], [5], [10], . Since Sd is one of the partners of the SWH pathway effector Yki [20]–[22], this raises the possibility that, via its effects on *sd* and *vg*, Notch could integrate with SWH to control epithelial proliferation. Taking a genetic strategy, we have found that the Notch pathway down-regulates *ex-lacZ*, the reporter of SWH activity, cell autonomously in wing pouch cells. This inhibition either operates through Yki or downstream of Yki. We further show that downregulating the activity of the Notch target Vg enhances the expression of the two Yki targets expanded and thread/DIAP1. Thus, Vg mediates, at least in part, the repressive activity of Notch in the wing pouch on Yki targets. We propose that by modifying the ratio between Sd, Vg and Yki, Notch signaling prevents Yki from activating its targets in the wing pouch, thus helping to co-ordinate tissue growth with patterning.

## Results and Discussion

### Notch activity in the wing pouch inhibits ex-lacZ expression

**Figure 1:**
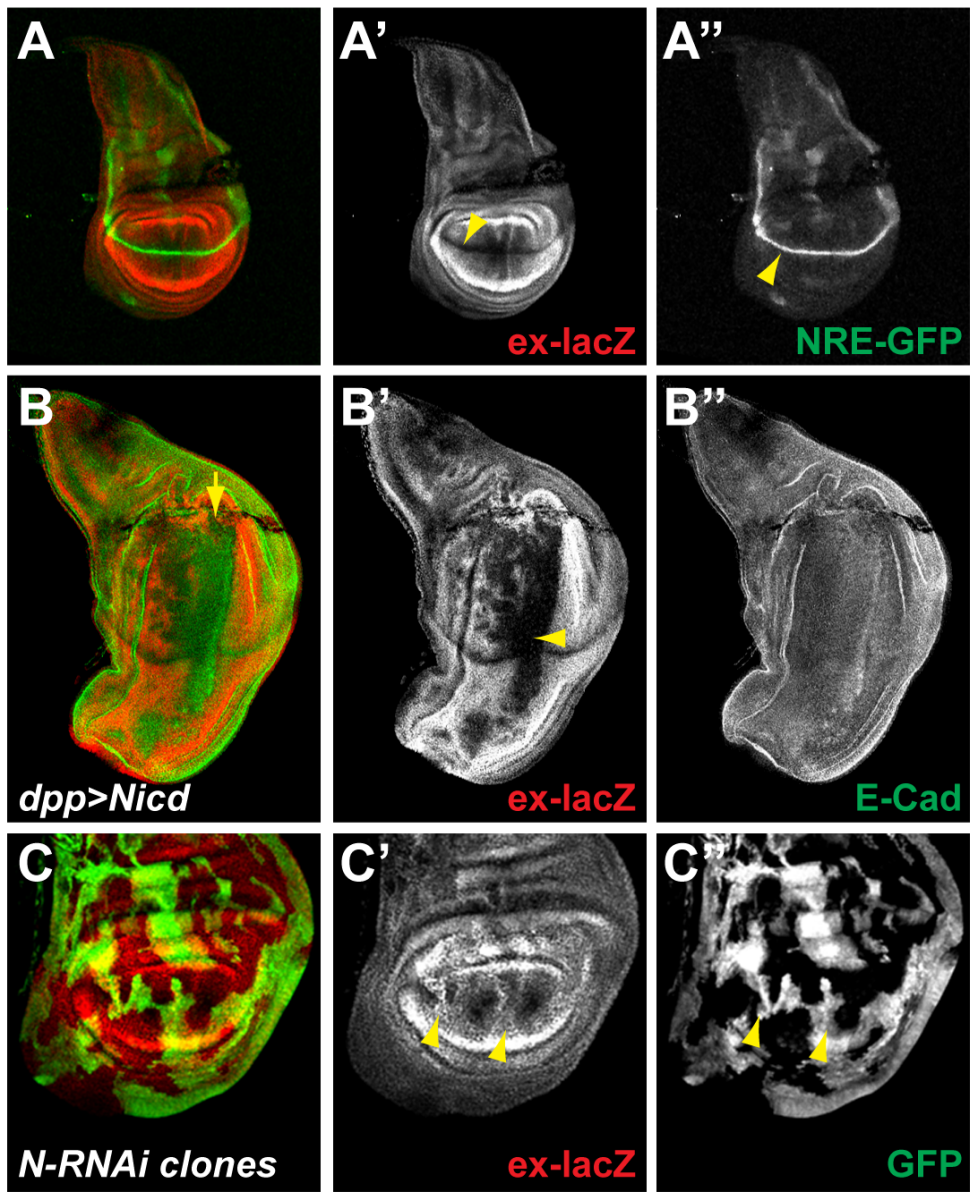
Notch signaling inhibits *ex* expression in the wing pouch. **A.** In third instar larval wing discs, the expression of the Yki target *expanded* monitored using the *ex^697^-lacZ* reporter line (ex-lacZ; red; A′) is strongest at the periphery and absent at the dorso-ventral boundary (D/V; yellow arrowhead). The activity of the Notch pathway monitored by the *NRE-GFP* reporter (green; A″) is highest at the D/V (yellow arrowhead). **B.** Over-expression of the active form of the Notch receptor (Nicd) in a stripe of cells along the antero-posterior (A/P) boundary of the wing disc using *dpp-Gal4* (along the line indicated by the yellow arrow), leads to the repression of *ex-lacZ* (red; B′; yellow arrowhead), while E-Cadherin levels are unaffected (E-Cad; green; B″). **C.** Inhibition of the Notch pathway in randomly generated clones overexpressing an RNAi against Notch (N-RNAi; positively marked by GFP; green; D″), leads to the upregulation of ex-lacZ in the pouch (red; D′; yellow arrowheads).

In order to investigate the possibility of cross talk between the Notch and Sav/Warts/Hippo (SWH) pathways, we first compared the expression pattern of *ex-lacZ*, a reporter of Yki activity, which reveals the places where SWH activity is lowest and NRE-GFP, which gives a direct read out of Notch activity (Fig. 1A). In the wing pouch these reporters direct expression in patterns that are complementary. Thus, *ex-lacZ* expression is completely absent from the dorso-ventral boundary where Notch activity, reported by *NRE-GFP*, is at its highest (Fig. 1A). Conversely, in late stage discs, *ex-lacZ* expression is higher in pro-vein regions where Notch activity (NRE-GFP) is low. Because *ex-lacZ* gives a mirror image of SWH activity, these results suggest that both Notch and SWH pathways are active together in the D/V boundary and are largely inactive in the pro-veins.

We then investigated the consequences of modulating Notch activity on the expression of *ex-lacZ* as an indicator of its effects on SWH pathway. Expression of Nicd, the constitutively active form of the Notch receptor, promoted a strong down-regulation of *ex-lacZ* in the wing pouch. This effect was stronger in the region surrounding the D/V boundary and weaker towards the periphery. Little down-regulation occurred outside the pouch (Fig. 1B). Conversely, when Notch activity was impaired, through RNAi mediated knock-down in randomly generated overexpression clones, *ex-lacZ* levels were up-regulated. This effect was also only evident within the wing-pouch (Fig. 1C). Notch activity is therefore necessary and sufficient for the inhibition of *ex-lacZ* in the wing pouch (Fig. 1B&C), suggesting that it contributes to the normal down-regulation of *ex-lacZ* at the D/V boundary.

### Notch acts at the level or downstream of Yki

**Figure 2:**
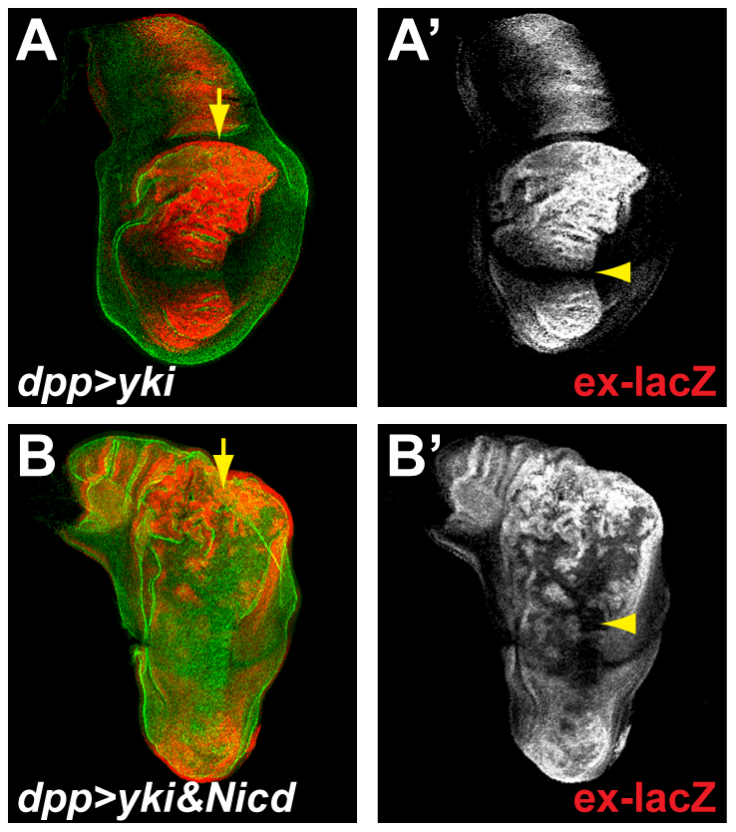
Notch acts at the level or downstream of yki. **A.** Over-expression of Yki along the A/P boundary of the wing disc using *dpp-Ga*l4 (along the line indicated by the yellow arrow), leads to the strong increase in expression of *ex-lacZ* in the pouch (red; A′), except at the D/V where Notch activity is highest (yellow arrowhead). **B.** When co-expressed with Yki (using dpp-Gal4; yellow arrow), Nicd imposes an inhibition of *ex-lacZ* expression in the pouch (red; B′; yellow arrowhead) but not at the periphery. E-Cadherin staining (green, A; B) outlines all cells.

In the wing pouch, *ex-lacZ* expression requires Yki. Therefore, to mediate the observed inhibition of *ex-lacZ* expression, Notch could either exert its actions upstream of Yki, by activating the SWH pathway, or downstream of Yki, by inhibiting Yki’s transcriptional activity. To determine which of these alternatives is correct, we assessed the consequences of Notch activity on ectopic Yki expression. When over-expressed in a stripe of cells along the A/P boundary, Yki was able to promote strong expression of *ex-lacZ* at the periphery of the wing pouch. Strikingly, the high levels of Yki were not able to force *ex-lacZ* expression at the D/V boundary where Notch activity is highest. These results suggest that the actions of Notch, ie *ex-lacZ* down-regulation, are epistatic to Yki (Fig. 2A). This was further verified when high levels of Yki were expressed together with high levels of Nicd. In this case, Nicd suppressed the *ex-lacZ* expression, demonstrating that it wins out over Yki in the wing pouch (Fig. 2B). However, at the periphery of the discs, Nicd was unable to modify the effects of Yki over-expression on *ex-lacZ* levels. Taken together these results suggest that Notch-mediated down-regulation of *ex-lacZ* occurs at the level or downstream of Yki.

### E(spl) and cut repressors are not required to pattern expanded expression

Since a major output of Notch pathway activity is the up-regulation of gene expression, we investigated whether any of the directly regulated Notch target-genes could be responsible for antagonizing Yki. Amongst the direct Notch targets identified in wing discs, several are predicted to encode transcriptional repressors. These include members of the HES family, E(spl)mβ, E(spl)m5, E(spl)m7, E(spl)m8 [23]–[27] and Deadpan (Dpn) [28], [29], as well as the homeodomain protein Cut. All of these proteins are normally expressed at high levels along the D/V boundary, in response to Notch activity, and hence are candidates to mediate the repression of *ex-lacZ.*

**Figure 3:**
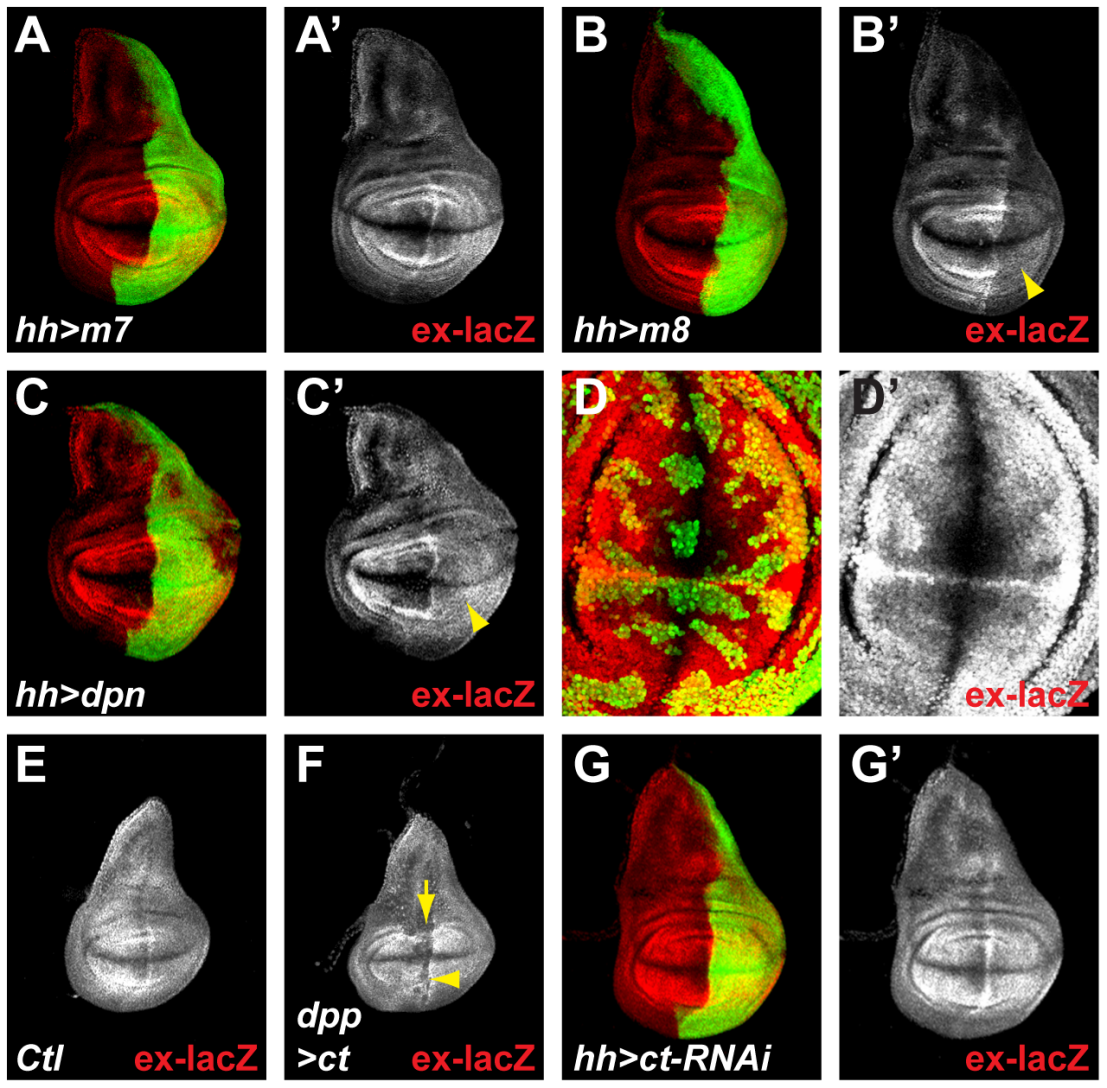
E(spl) and Cut repressors do not mediate the effects of Nicd on *ex-lacZ* expression. **A–C.** When over-expressed in the posterior compartment using *hh-Gal4* (marked by GFP; green), the individual HES factors have different effects on *ex-lacZ* (red; A′, B′, C′). While E(spl)m*7* (and others see text; m7; A) has no effect on *ex-lacZ*, E(spl)m8 (m8; B) and Deadpan (dpn; C) induce a strong down-regulation of *ex-lacZ* (yellow arrowhead; B′, C′). **D.** MARCM clones, marked positively by GFP (green), which are homozygous mutant for all *E(spl)* genes (*Df(3R)E(spl)[Gro^b32.2^*]) and express a strong RNAi against *dpn* show normal expression of *ex-lacZ* (red; D′). **E–F.** Early third instar larval wing discs showing *ex-lacZ* expression. Over-expression of Cut (ct) along the A/P boundary of the wing disc using *dpp-Gal4* (along the line indicated by the yellow arrow) inhibits *ex-lacZ* expression (F; yellow arrowhead) compared to controls (E). **G.** Over-expressing an RNAi construct against *ct* in the posterior compartment using *hh-Gal4* (marked by GFP; green) has no effect on *ex-lacZ* expression (red; G′).

Over-expression of E(spl)mβ, E(spl)m5 or E(spl)m7 repressors had no effect on *ex-lacZ* expression (Fig. 3A and data not shown). In contrast, over-expression of either E(spl)m8 or of Dpn resulted in a robust down-regulation of *ex-lacZ* (Fig. 3B&C). The effect differed slightly from that from Nicd expression, in that *ex-lacZ* expression was not completely abolished and low levels persisted throughout the wing pouch domain (compare Fig. 1B with Fig. 3B&C). These results indicate that a subset of the HES bHLH proteins have the capability to repress *ex-lacZ*, and hence are candidates to antagonize Yki. Previous experiments have demonstrated that the E(spl)bHLH genes and *dpn* have overlapping functions, especially at the D/V boundary [28]–[30]. Therefore to determine whether these factors normally contribute to the repression of *ex-lacZ*, it was necessary to eliminate all of the *E(spl)bHLH* genes in combination with *dpn*. To achieve this we expressed a potent RNAi directed against *dpn* in MARCM clones that were homozygous mutant for a deficiency removing the entire *E(spl)* complex (details in Materials and Methods). No derepression of *ex-lacZ* was detectable in such clones, suggesting that none of the *E(spl)bHLH/dpn* genes can account for the repression of *ex-lacZ* at the D/V boundary or in the wing pouch (Fig. 3D). Therefore even though E(spl)m8 and Dpn expression is sufficient for *ex-lacZ* repression, they do not appear to be essential in the context of the wing pouch.

An alternative candidate was Cut, which encodes a transcriptional repressor and is expressed at the D/V boundary in response to Notch signaling [2], [10], [31], [32]. Similar to some of the HES genes, over-expression of Cut promoted a down-regulation of *ex-lacZ* (Fig. 3E&F). This was most clearly evident at early developmental stages because Cut induced a strong epithelial delamination at later stages, confounding the interpretation. However no up-regulation of *ex-lacZ* was detectable when Cut function was ablated, using RNAi, even though Cut levels where efficiently reduced (Fig. 3G & data not shown). Thus, as with the HES genes, Cut is capable of inhibiting *ex-lacZ* expression but does not appear to be essential for the regulation of *ex* under normal conditions in the wing pouch.

### Vg mediates the effects of Notch on ex-lacZ

Recent studies have demonstrated that, in the absence of Yki, several SWH target genes are kept repressed by Sd, the DNA-binding partner of Yki. This so-called “default repression” requires Tgi, an evolutionarily conserved tondu domain containing protein, which acts as a potent co-repressor with Sd [33], [34]. There is no evidence that *Drosophila tgi* is a target of Notch in the wing disc, making it an unlikely candidate to mediate the inhibitory effects on Yki-mediated *ex-lacZ* expression. However, *vg*, which encodes another Sd binding-partner with a tondu domain [18], [19], is directly regulated by Notch in the wing pouch [9]–[11]. We therefore hypothesized that Vg could mediate the effects of Notch on Yki function and *ex-lacZ* down-regulation.

**Figure 4:**
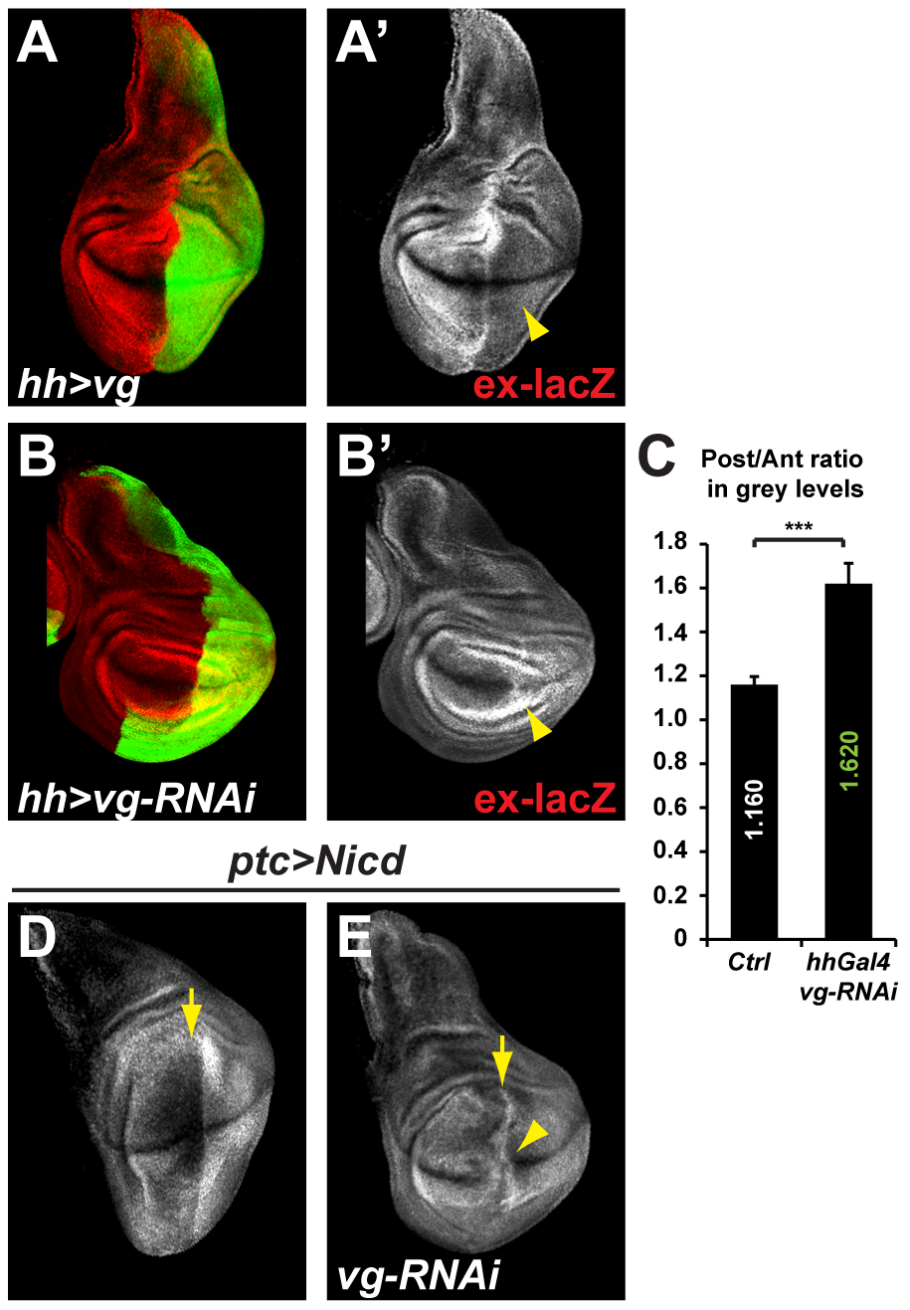
Vg mediates in part the effects of Nicd on *ex-lacZ* expression. **A–B.** Effects of over-expressing the Notch target *vestigial* (vg; A) or RNAi constructs against *vg* (B) in the posterior compartment using *hh-Gal4* (marked by GFP; green) on *ex-lacZ* expression (red; A′&B′). Vg over-expression and RNAi leads to the inhibition and up-regulation of *ex-lacZ* respectively (A′; B′; yellow arrowheads). **C.** Quantification of the average ex-lacZ intensity ratios between equivalent area picked in the posterior and anterior compartments in several discs. While the ratio is at 1.160 in control discs reflecting a slightly higher expression in the posterior compartment, this ratio rises to 1.620 in experimental discs showing that *ex-lacZ* expression is higher in *vg* depleted compartments. Standard error to the mean is shown; unpaired two-tailed student t-test was performed showing significance with p value = 0.0003 (***). **D–E.** While the overexpression of Nicd at the antero-posterior boundary (along the line indicated by the yellow arrow, using the ptc-Gal4 driver), leads to an inhibition of *ex-lacZ* (white; D), co-expressing an RNAi against *vg* suppresses this effect, and *ex-lacZ* expression is found throughout the ptc domain including the D/V boundary (white; E; yellow arrowhead).

In agreement with the hypothesis, when Vg was over-expressed it strongly inhibited *ex-lacZ* expression in the pouch and promoted a modest overgrowth of the tissue (Fig. 4A). This overgrowth is somewhat puzzling since it appears that Yki activity, as monitored by *ex-lacZ*, is lowered in the presence of excess Vg. How over-expressed Vg triggers overgrowth remains poorly understood, but has been proposed to involve a cross-talk with the wg pathway [3], [10], [11]. More recently, it has been shown that the expansion of the pouch region is achieved by Vg activating transiently and non-autonomously Yki in cells not expressing Vg. These cells are then recruited to become wing pouch cells and turn on *vg* expression [35]. This model predicts a wave of Yki activation around Vg positive cells. Therefore, the overgrowth seen when Vg is over-expressed, could be due to a non-autonomous effect where more cells are recruited as pouch cells at the expense of more peripheral cells. Alternatively, Vg could promote proliferation of the pouch cells by an as yet unidentified mechanism, independent of Yki.

Conversely to over-expressed Vg inhibiting *ex-lacZ* expression, lowering the levels of *vg* using RNA interference in the whole posterior compartment resulted in a significant up-regulation of *ex-lacZ* (Fig. 4B). Vg knock down has proven difficult to achieve in small populations of cells, due to their elimination from the wing pouch as documented previously [21], probably by cell competition. Thus, unlike the other factors tested, Vg is required for the repression of *ex-lacZ* in the wing pouch. We further show that, co-expressing with NICD a *vg RNAi* transgene in the patched domain, suppresses the NICD mediated *ex-lacZ* repression in the wing pouch (4C&D). Taken together, these results suggest that Vg mediates the repressive effects of Notch on *expanded* expression.

### Vg prevents Yki targets expression in the pouch

If the involvement of Vg downstream of Notch is a general mechanism for cross-talk between Notch and Yki, other targets of the Sd-Yki complex should be inhibited by Notch in a similar manner to ex-lacZ. However, apart from *expanded*, all other known Yki targets in the wing pouch, such as *thread/DIAP1*, *diminutive/myc,* and *Cyclin E* are also direct Notch targets [5], [20]–[22], [36]–[42]. Their final expression patterns are therefore a reflection of the balance between different transcriptional inputs, in particular Notch and Yki. Our model predicts that Notch could have a dual effect on the expression of genes: a positive direct effect through the NICD/Su(H) complex when bound in their promoters, but also a negative effect through the induction of Vg, which prevents the positive effect of Yki on Sd bound promoters.

In agreement with this model, *thread/DIAP1* and *diminutive/myc*, two well established Yki targets in wing discs [21], [22], [38], which are normally refractory to Notch mediated activation in the centre of the pouch, become susceptible to Nicd when Vg or Sd levels are lowered through RNAi [5].

Focusing on *DIAP1*, we thought to separate the Notch and Yki direct inputs on transcription by isolating the Hippo pathway Responsive Elements (HREs) from any potential Notch Responsive Elements (NREs). *IAP2B2C-lacZ* is a previously described DIAP1-HRE driving lacZ reporter expression [21] that does not contain any NRE, at least based on our Su(H) ChIP data and bio-informatics prediction of Su(H) binding sites [5]. Our model predicts that this IAP2B2C-lacZ reporter should be inhibited by Vg.

**Figure 5:**
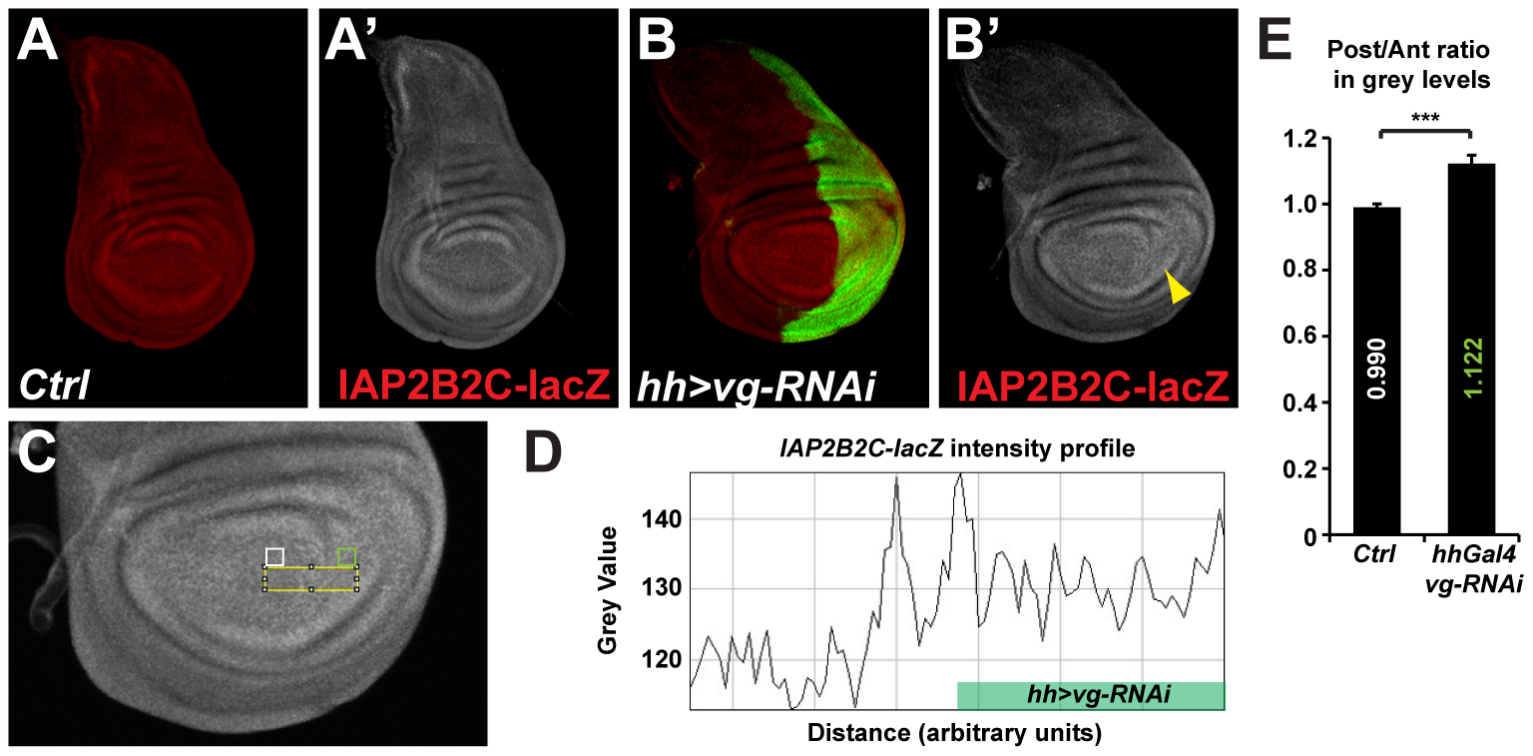
Vg represses *th/DIAP1* expression in the wing pouch. **A–B.** Expression of the SWH pathway specific *th/DIAP1* reporter *IAP2B2C-lacZ* (red; A′&B′). While IAP2B2C-lacZ are uniform between the anterior and posterior of the pouch in control discs (A), a slight increase in the posterior compartment could be detected in discs where vg activity is lowered by RNAi only in the posterior compartment (B; marked by GFP; green; yellow arrowhead). **C–E.** Quantification of the posterior compartment increase of *IAP2B2C-lacZ* expression after vg RNAi knock-down. **C.** Higher magnification of the image in B′ showing *IAP2B2C-lacZ* expression in *hh-Gal4 vg-RNAi* expressing discs. **D.** Example of the profile of grey levels (reflecting IAP2B2C-lacZ levels), along the yellow box indicated in C. Levels are higher in the posterior compartment expressing the vg-RNAi than in anterior, except for effects at the boundary that are not understood. **E.** Quantification of the average IAP2B2C-lacZ intensity ratios between equivalent area picked in the posterior (green box in C) and anterior (white box in C) compartments in several discs. While the ratio is at 0.990 in control discs reflecting similar expression levels between the posterior and anterior compartments, this ratio rises to 1.122 in experimental discs showing that *IAP2B2C-lacZ* expression is 12% higher in the *vg* depleted compartment. Standard error to the mean is shown; unpaired two-tailed student t-test was performed showing significance with p value = 0.0006 (***).

In control wing discs, we confirm that *IAP2B2C-lacZ* is expressed at uniform low levels with a slight increase at the periphery of the pouch (Fig. 5A), where Vg protein levels have been shown to fade [10], [18]. The D/V boundary expression of *DIAP1* is not reported by *IAP2B2C-lacZ* confirming that the NRE is absent in this reporter.

We then lowered Vg levels using moderate RNAi knocked down in the whole posterior compartment using the *hh-Gal4* driver (Fig. 5B; as mentioned before, since Vg is required for pouch identity, strong *vg* knock down results in delaminating cells that are difficult to interpret). In this experimental set-up, the posterior compartment is smaller than normal, and *vg* knock-down induced a 12% up-regulation of *IAP2B2C-lacZ* expression when compared to *IAP2B2C-lacZ* levels in the anterior control compartment (Fig. 5C–E), demonstrating that Vg has a negative effect on this reporter activity (there was no difference in *IAP2B2C-lacZ* expression between the anterior and posterior compartment in the pouch region of control discs; Fig. 5A&E). We note that *IAP2B2C-lacZ* expression was up-regulated in a small stripe of cells in the anterior compartment just at the boundary with *vg* depleted cells (Fig. 5D). This region was excluded from our quantifications, but suggests that the *IAP2B2C-lacZ* reporter fragment could be sensitive to a non-autonomous input acting around the boundary of cells with different Vg levels.

It appears therefore, that at least for the two Yki targets *ex-lacZ* and *IAP2B2C-lacZ*, Vg inhibits their expression in the wing pouch. Previous studies reported independent roles of Vg and Yki on the activation of their targets, and could appear to contradict this newly described inhibitory role of Vg on Yki targets. However, in these studies, the authors demonstrated that Vg and Yki do not require each other to promote wing pouch cell survival and to activate their respective targets (Pan Dev Cell 2008), which does not rule out any negative cross regulation, as shown in this report.

### A complex network between Notch, Yki, and Vg

Our analysis brings therefore new evidence of the central role of Vg in the complex network regulating wing disc growth [43], adding a new level of complexity in its interaction with the SWH pathway effector Yki.

Thus, Notch induced expression of Vg could give rise to an Sd-Vg repressive complex that prevents expression of Yki targets [44], [45]. In situations where SWH signaling is lowest, Yki levels may be sufficiently high to overcome this repression. This suggests that in the wing pouch, Notch and SWH would act co-operatively rather than antagonistically.

Outside of the pouch, at the wing disc periphery, *sd* and *vg* expressions are not promoted by Notch activity. Furthermore, other binding partners for Yki, such as Homothorax are expressed there and might substitute for Sd to control the expression of Yki targets in a way similar to what has been described in the *Drosophila* eye [46]. The differential expression of these transcription factors in the disc could explain why Notch only has an inhibitory effect on Yki targets in the wing pouch. Furthermore, it is also worth noting that Notch has very different effect outside of the pouch, where it promotes Yki stabilization non-autonomously via its regulation of ligands for the Jak/Stat pathway [47].

In summary, our evidence demonstrates that Notch activity can inhibit Yki under circumstances where Yki acts together with Sd. It does so by promoting the expression of Vg, a co-factor for Sd, counteracting the effects of Yki. This cross talk potentially extends to mammalian systems as the active form of NOTCH1, NICD1 promotes the up-regulation of VGLL3 (a human homologue of vg) in MCF-10A breast cancer derived cells [48]. Thus, similar mechanisms may also be important in mediating interactions between the NOTCH and SWH pathways in human diseases.

Because the end-point of SWH pathway activity is to prevent Yki function, the inhibitory effects of Notch on Yki could provide an explanation for those cellular contexts where the two pathways act co-operatively, as at the D/V boundary in the wing discs. Similar co-operative effects have been noted in the *Drosophila* follicle cells. However, in this case it is the SWH activity that is involved in promoting the expression of Notch targets [49], [50]. In other contexts, such as the mouse intestine, accumulation of Yap1, the mouse Yki homolog, and therefore inhibition of the SWH promotes Notch activity [51], [52]. These examples demonstrate that the interactions between Notch and the SWH are highly dependent on cellular context. Our results suggest that some of these differences may be explained by the nature of the target genes that are regulated and by which Yki co-operating transcription factors are present in the receiving cells.

## Materials and Methods

### Fly stocks

*NRE-GFP (86Fb; {GreenRabbit-ins})* [53], *P{lacW}ex^697^* (gift from Nic Tapon), and IAP2B2C-lacZ (gift from Duojia Pan) [21] were used to monitor the activity of the Notch and SWH pathways respectively.

*UASt Nicd[79.2], UASt Nicd[MH3], UASt yki* (gift from Nic Tapon), *UASt E(spl)mb, UASt E(spl)m5, UASt E(spl)m7, UASt E(spl)m8, UASt dpn* (gift from Harald Vaessin), *UASt cut* (gift from Joel Silber), *UASt vg* (gift from Joel Silber) transgenes carrying flies were crossed to flies carrying the wing discs drivers *patched[559.1]-Gal4*, *dpp[disc]-Gal4*, or *hh-Gal4* (gift from Nic Tapon) at 25C to over-express the corresponding gene products either in a stripe of cells at the anterior-posterior boundary (*ptc-Gal4* and *dpp-Gal4*), or in the whole posterior compartment (*hh-Gal4*).

The *ct, dpn, Notch*, and *vg* gene products were knocked-down using flies carrying the following UAS RNAi transgenes, *P{TRiP.HMS00924}attP2, P{KK101812}VIE-260B, P{UAS-N.dsRNA.P}14E, P{GD1558}v16896* respectively and crossed with the *hh-Gal4* driver at 30C.

MARCM clones of cells null mutant for all bHLH coding *E(spl)* genes and with a strong RNAi against *dpn* were generated crossing the two following lines:

- hsFLP122, UASt GFPn, tub-Gal4;; FRT82B tub-Gal80

- P{KK101812}VIE-206B; FRT82B Df(3R)E(spl)[Gro^b32.2^] [gro+]

*E(spl)[Gro^b32.2^]* is a deficiency covering the whole *E(spl)* complex with a breakpoint close to *groucho.* The chromosome carries a *groucho* [gro+] construct which fully rescues *groucho* function [25]. Progeny larvae from this cross were heat-shocked for 1h at 37C to induce MARCM clones positively marked with GFP. The larvae were subsequently maintained at 30C to ensure the most efficient RNAi effect on *dpn*.

### Immunofluorescence

Immunostainings were performed according to standard protocols. Antibodies used were: mouse anti-b-Galactosidase (Developmental Studies Hybridoma Bank - DSHB 40-1a; 1/25), rat anti-ECadherin (DSHB DCAD2; 1/25), rabbit anti-GFP (Molecular Probes A6455; 1/2000).

Images were acquired on a Leica SP2 confocal microscope and analysed using Adobe Photoshop or the FIJI ImageJ package.
